# Regulation of antitumor immunity by inflammation-induced epigenetic alterations

**DOI:** 10.1038/s41423-021-00756-y

**Published:** 2021-08-31

**Authors:** Michael Karin, Shabnam Shalapour

**Affiliations:** 1 Department of Pharmacology, School of Medicine, University of California San Diego, La Jolla, CA USA; 2 Laboratory of Gene Regulation and Signal Transduction, Department of Pharmacology, School of Medicine, University of California San Diego, La Jolla, CA USA; 3 Department of Cancer Biology, University of Texas MD Anderson Cancer Center, Houston, TX USA

**Keywords:** Inflammation, Epigenetics, Antitumor immunity, Immunotherapy, Cancer, Tumour immunology, Cell signalling

## Abstract

Chronic inflammation promotes tumor development, progression, and metastatic dissemination and causes treatment resistance. The accumulation of genetic alterations and loss of normal cellular regulatory processes are not only associated with cancer growth and progression but also result in the expression of tumor-specific and tumor-associated antigens that may activate antitumor immunity. This antagonism between inflammation and immunity and the ability of cancer cells to avoid immune detection affect the course of cancer development and treatment outcomes. While inflammation, particularly acute inflammation, supports T-cell priming, activation, and infiltration into infected tissues, chronic inflammation is mostly immunosuppressive. However, the main mechanisms that dictate the outcome of the inflammation-immunity interplay are not well understood. Recent data suggest that inflammation triggers epigenetic alterations in cancer cells and components of the tumor microenvironment. These alterations can affect and modulate numerous aspects of cancer development, including tumor growth, the metabolic state, metastatic spread, immune escape, and immunosuppressive or immunosupportive leukocyte generation. In this review, we discuss the role of inflammation in initiating epigenetic alterations in immune cells, cancer-associated fibroblasts, and cancer cells and suggest how and when epigenetic interventions can be combined with immunotherapies to improve therapeutic outcomes.

## Introduction

The traditional definition of inflammation, established by the nineteenth and twentieth century pathologists, is the activation of innate immune cells by damage- and microbe-associated molecular patterns, and inflammation is indicated by macrophage and neutrophil recruitment and manifestation of the five cardinal signs: rubor (redness), calor (heat), tumor (swelling), dolor (pain), and functio laesa (loss of function). In contrast, T-cell inflammation, which is used to describe tumors as “hot” or “cold”, is a new term coined by tumor immunologists. Although pathogenic T cells are important components of many chronic inflammatory diseases, their presence within a tumor does not necessarily reflect classical inflammation, and their number or density does not always correlate with the outcome of immunotherapy. Moreover, fibrosis, a frequent consequence of chronic inflammation, may directly or indirectly affect T-cell recruitment and responses. Finally, what we refer to as epithelial or cancer cell-intrinsic inflammation pertains to the inflammatory signaling pathways that are activated in these cells in response to stress, pathogen exposure or oncogene activation. In summary, inflammation can profoundly modify gene expression profiles and cause epigenetic changes that can be long lasting, resulting in memory development not only in adaptive immune cells but also in innate immune cells (trained immunity) and epithelial cells, as well as perturbed tissue homeostasis [1–3]. This review will detail the interactions between inflammation and epigenetics and their effects on tumor immune evasion, focusing on cancer cells and cellular components of the tumor microenvironment. We also discuss how these connections can be targeted to improve cancer prevention and treatment.

## Role of inflammation in initiating epigenetic alterations

Histone modifications, DNA methylation and noncoding RNAs (ncRNAs) have emerged as master regulators of gene expression. DNA is organized into structures called nucleosomes, which contain DNA wrapped around an octamer of histone proteins. Each histone has a long tail that can be posttranslationally modified by phosphorylation, acetylation, methylation, SUMOylation, and ubiquitinoylation, and these modifications affect chromatin compaction and gene transcription [4]. Lysine acetylation neutralizes positive charges, whereas phosphorylation adds negative charges, resulting in reduced chromatin compaction and enhanced transcription [5, 6]. Histone acetyltransferases (HATs), such as p300/CBP, catalyze the acetylation of specific lysine residues in the histone tail. p300/CBP, in particular, cooperates with the Trithorax group of proteins to acetylate histone 3 at lysine 27 (H3K27), a modification that ultimately promotes the activation of specific genes during development [7]. Histone deacetylases (HDACs) antagonize the effects of HATs by removing acetyl groups from lysine residues in the histone tail to result in transcriptional repression. HDACs include classical family members, which have a zinc-dependent catalytic site, and “silent information regulator 2-related proteins” (sirtuins) [8], which are NAD^+^ dependent. Methylation of histones can be repressive or activating, depending on the residue being methylated. Trimethylation of histone H3 at lysine 27 (H3K27me3) mediated by the histone methyltransferase (HMT) enhancer of zeste homolog 2 (EZH2), a member of the polycomb repressive complex 2, is associated with transcriptional repression [9]. These modifications influence the recruitment of proteins, including transcription factors (TFs), and interfere with the binding of protein complexes that modulate chromatin density and accessibility [10]. DNA methylation at cytosine residues adjacent to guanine residues (CpG sites) occurs frequently throughout the genome. This reaction is catalyzed by DNA methyltransferases (DNMTs), such as DNMT3a and DNMT3b, which are responsible for de novo methylation of unmethylated DNA. In addition to preventing TFs from binding to promoters, DNA methylation can lead to the binding of DNA methyl-binding domain-containing proteins, which further inhibit gene transcription by recruiting repressive complexes containing HDACs and HMTs [11].

The immune system can support or suppress cancer initiation and progression. Epigenetic alterations, which are changes in chromatin structure and gene expression without DNA sequence changes, play a fundamental role in tumorigenesis through silencing tumor suppressor genes or activating oncogenic signaling. Epithelial cells and cancer cells affected by chronic inflammation undergo changes in DNA methylation and histone modifications, indicating that inflammation results in epigenetic alterations. Furthermore, epigenetic modifications occur during immune cell differentiation, activation and particularly memory development and have pronounced effects on the production of inflammatory cytokines. Moreover, cancer cells were observed to epigenetically silence immune-related genes to evade immune recognition and rejection.

## Molecular mechanisms underlying inflammation-induced epigenetic alterations

There are a substantial amount of epidemiological data, as well as in vivo and vitro studies, that confirm a connection between inflammation and epigenetic alterations [12–17]. One mechanism by which inflammation can affect the epigenome is by altering the metabolic state of the cell. Moreover, the effects of diets or caloric restriction on longevity, cancer, and the immune system have been associated with the histone modification abilities of these factors [18–20]. The metabolism of immune cells is altered when they are activated, and in turn, activated immune cells modulate metabolic processes in the surrounding tissue [21]. Altered DNA methylation, histone modifications, and dysregulated miRNA expression have each been linked to impaired metabolic functions in organs actively involved in glucose/lipid homeostasis [22]. Such alterations have also been found in obese and type 2 diabetic (T2DM) individuals [23, 24], in whom they were shown to support chronic inflammation and cancer development. Hepatic steatosis is associated with signal transducers and activators of transcription (STAT) 3 activation, and activated STAT3 can regulate metabolism by inducing aerobic glycolysis and decreasing mitochondrial activity [25]. Many epigenetic enzymes require metabolic intermediates for their activity [26]. S-adenosyl methionine (SAM) is a required cofactor for DNMTs and HMTs, whereas S-adenosyl homocysteine is an inhibitor. SAM is produced by 1-carbon metabolism and through dietary consumption of methyl group donors (i.e., folate). Recently, it was shown that cancer cells disrupt methionine metabolism in CD8^+^ T cells by consuming methionine and outcompeting T cells for this amino acid through high expression of the methionine transporter SLC43A2. Lower intracellular levels of methionine and the methyl donor SAM in CD8^+^ T cells were shown to result in loss of H3K79me2, which decreased STAT5 levels and impaired T-cell immunity [27].

HDMs and ten-eleven translocation methylcytosine dioxygenase proteins use alpha-ketoglutarate, a TCA cycle intermediate, as a cofactor [28, 29]. Acetyl–CoA (Ac-CoA) is produced by glucose metabolism and is an important cofactor for HATs. NAD^+^ is an essential cofactor for the HDAC SIRT1, and SIRT1 has been linked to metabolic disorders. In this regard, lactate dehydrogenase A (LDHA) is induced in activated T cells to support aerobic glycolysis and promote IFNγ production. LDHA maintains high concentrations of Ac-CoA to enhance histone acetylation and *Ifng* transcription [30].

Moreover, the introduction of double-stranded DNA breaks, which accumulate during aging and chronic inflammation, results in recruitment of the epigenetic modulators SIRT1, EZH2, DNMT1, and DNMT3b to sites of DNA damage [9, 17, 31, 32].

## Epigenetic regulation of tumor microenvironment components

### Innate immune cells, macrophages, and antigen-presenting cells

The immediate response to infection, cell damage or perturbation of tissue structure is mounted by the innate immune system, which consists of epithelial cell barriers and immune cells, including macrophages, neutrophils, dendritic cells (DCs), and natural killer (NK) cells. This response results in classical inflammation, consisting of the five signs described above [33–35]. Tissue-resident macrophages, DCs, and mast cells usually initiate acute inflammatory responses upon recognition of pathogen- or damage-associated molecular patterns by Toll-like and Nod-like receptors (NLRs) via production of chemoattractants, cytokines, and inflammatory mediators, which induce the cardinal signs of inflammation and recruit innate immune cells to the site of injury or infection [36]. These cells, particularly DCs, differentiate after taking up antigens, which can then be presented to adaptive immune cells, leading to the activation and recruitment of those cells. At this point, the inflammatory response may be sustained by adaptive immune cells, which also maintain immune memory. Epigenetic programs control the molecular mechanisms that regulate immunological memory. Recently, it was shown that epigenetic programming can also regulate a memory-like phenotype in innate immune cells. The initial inflammatory response produced by innate cells also affects epithelial, endothelial, and mesenchymal cells in the surrounding microenvironment and contributes to chromatin reprogramming in those cells [2, 3, 37]. Cytokines activate specific signaling pathways in epithelial and mesenchymal cells that result in the production of more chemokines and cytokines as well as epithelial or mesenchymal cell proliferation. Reactive oxygen species and reactive nitrogen species damage lipids, proteins, and DNA within affected epithelial cells, causing activation of various stress response pathways. The main TFs activated by these response pathways are members of the AP-1 and CREB families of bZIP proteins, STATs such as STAT1 and STAT3, and NF-κB family members, which also regulate and are frequently regulated by HDACs and HATs. Moreover, epigenetic processes play fundamental roles in the development and differentiation of innate immune cells, although knowledge concerning these roles is limited compared to that on the roles that affect the development and differentiation of adaptive immune cells. Two examples are the roles of epigenetic factors in macrophage polarization [31, 38] and the development of a memory-like phenotype in innate immune cells, which are attributed to epigenetic reprogramming [1]. Active and repressive chromatin marks are known to regulate the expression of key cytokine genes according to the macrophage polarization phenotype [39]. Another study suggested that conversion from the M2-like to the M1-like phenotype is controlled by DNMT3b in obesity [40]. Specifically, in response to stearic acid, DNMT3b expression is induced in macrophages and followed by binding of DNMT3b to the promoter of peroxisome proliferator-activated receptor gamma 1 (PPARγ1), a key regulator of macrophage polarization. This leads to hypermethylation of the *Pparγ1* promoter and likely contributes to the phenotypic switch[40].

Moreover, in macrophages, ornithine decarboxylase, which is an essential enzyme in polyamine synthesis, controls the antimicrobial M1-like response during *Helicobacter pylori* and *Citrobacter rodentium* infections by regulating histone modifications at both the enhancers and promoters of proinflammatory cytokine genes[41]. Lack of glucose availability reduces the cytoplasmic NADH:NAD^+^ ratio and promotes binding of the NAD(H)-sensitive transcriptional corepressor CtBP to p300, which in turn blocks the binding of p300 and NF-κB to proinflammatory gene promoters through regulation of p65/RelA acetylation[42]. NAD^+^ levels also modulate sirtuin (e.g., SIRT1 and SIRT2) activity, which affects NF-κB p65/RelA activation via deacetylation of p65/RelA at lysine 310 [43, 44]. These modifications, which also affect NLRP3 inflammasome activity, demonstrate how metabolites regulate innate immunity through epigenetic mechanisms[31, 44].

### Adaptive immune cells: T cells and B cells

Adaptive immune responses are highly specific and provide long-term protection due to immune memory that enables recall responses. Naïve B and T lymphocytes expressing antigen receptors (BCR and TCR, respectively) reside in the lymph nodes. Antigen-presenting cells of the innate immune system activate lymphocytes specific to the presented antigen, triggering proliferation of the lymphocytes and their differentiation into effector cells. Naïve CD4^+^ and CD8^+^ T cells recognize antigens that are presented by major histocompatibility complex (MHC) II or I, respectively. When naïve CD4^+^ T cells encounter MHC II-bound antigens, a cascade of events is initiated, including activation and relocalization of various TFs. These changes in gene expression determine whether naïve T helper (h) cells become Th1, Th2, Th17, or Treg cells. Although TFs are pivotal in T-cell fate determination, more stable control of these transcriptional alterations is achieved via epigenetic mechanisms, including DNA methylation, histone modifications, ncRNAs, and chromatin remodeling[45]. Naïve CD4^+^ T cells and Th2 cells are characterized by the presence of H3K27me3, a repressive histone modification, at the *Ifng* locus, whereas Th1 cells show increased levels of H3K4me2, a histone marker associated with actively transcribed chromatin, at the same locus[46]. Memory CD8^+^ T cells have increased levels of diacetylated histone H3 at the *Ifng* locus compared to naïve CD8^+^ T cells, with the degree of gene-specific acetylation directly proportional to the efficiency of the recall response[47]. Moreover, treatment of T-cell lines with the DNMT inhibitor 5-azacytidine was shown to lead to the production of IL-2 and IFN-γ [48, 49]. Binding of T-bet to the *Ifng* promoter leads to displacement of HDACs and recruitment of HATs, thereby permitting *Ifng* transcription and contributing to Th1 cell differentiation[50]. With regard to the Th17 cell lineage, STAT3 has been shown to bind at the *Il17a* and *Il17b* promoters and induce histone acetylation, thereby stimulating transcription of these genes[51]. B cell proliferation and differentiation are also controlled by histone modifications, as suggested by the ability of the HDAC inhibitor (HDACi) panobinostat to control B cell numbers in a lupus-prone mouse model [52, 53]. Moreover, EZH2 is required for germinal center formation, B cell differentiation regulation, plasma cell metabolism and antibody production [54, 55]. Nutritional and energy states also influence the epigenome of immune cells. Variable levels of different metabolites affect the chromatin state and DNA methylation by influencing the enzymatic activity of chromatin modifiers and DNMTs. Increased glycolysis and β-oxidation in immune cells lead to elevated intracellular levels of Ac-CoA, which, when used as a group donor by HATs, causes a relaxed, more transcriptionally active chromatin state[53]. Moreover, EZH2 epigenetically regulates T-cell differentiation and Treg cell function, and its modulation in T cells was shown to improve the efficacy of immune checkpoint therapy (ICT) [56–58].

Chromatin organization also has a central role in T-cell exhaustion. In addition to the chronic LCMV infection model, tumor models have confirmed the role of epigenetic modifications in T-cell exhaustion. ATAC-seq showed that a consistent chromatin remodeling program dominated the conversion of effector T cells into exhausted T cells, which was absent from the memory T-cell formation program[59]. In fact, there are two discrete exhaustion-associated chromatin states, one in which T-cell exhaustion can be reversed and one in which T cells become permanently exhausted[59].

### Fibroblasts

Tissue-resident fibroblasts are emerging as one of the key cell types that regulate local immune cell responses during persistent infections, inflammation, and cancer [60–62]. Fibroblasts are heterogeneous cells with functionally distinct populations. Interestingly, their widely ranging abilities to regulate immune responses seem to vary in a context-dependent manner. Moreover, their phenotypes differ according to their tissue of origin, the disease and the type of inflammation. The immunological properties of fibroblasts are also diverse, ranging from maintenance of the inflammatory environment in chronic inflammation, immune cell recruitment, and activation of a robust immune response to immunosuppression, immune cell exclusion, and encapsulation of infected cells [60, 63–66]. Cancer-associated fibroblasts (CAFs) can recruit adaptive and innate immune cells to the tumor microenvironment by producing chemokines and cytokines, such as CC-chemokine ligand (CCL) 2, CCL5, CXC-chemokine ligand (CXCL) 8, CXCL12, chitinase 3-like protein 1 (CHI3L1) and IL-6 [61, 62, 67–69]. CAFs can contribute to T-cell dysfunction via antigen-dependent deletion, in which CAFs present antigens via MHC class I while engaging their PD-L1/2 and FAS ligands with PD-1 and FAS on T cells, respectively. CAFs also actively exclude T cells from tumors by producing transforming growth factor-β and CXCL12 [61, 67, 70]. Finally, CAFs contribute to the immunosuppressive tumor environment by retaining and recruiting regulatory T cells (Tregs) and IgA^+^ cells, polarizing macrophages toward a suppressive phenotype, and reducing the ability of DCs to present antigens and activate adaptive immunity [61, 68]. Moreover, hypoxia induces chemokine expression by prostate cancer fibroblasts [67] and epigenetic reprogramming of normal fibroblasts in breast cancer, resulting in a proglycolytic, CAF-like transcriptome[71]. These CAFs exhibit a metabolic shift toward lactate and pyruvate production and fuel biosynthetic pathways of cancer cells[71]. Moreover, lactate-mediated epigenetic reprogramming regulates the formation of human pancreatic CAFs and regulates their cytokine expression[72]. CAFs generally exhibit a profound alteration in gene expression compared to tissue-resident fibroblasts, which is attributed to the epigenetic changes in CAFs during cancer development, as no genetic alterations are usually found in CAFs isolated from solid tumors[73–75]. However, in childhood and infant acute lymphoblastic leukemia, bone marrow mesenchymal stem cells were shown to harbor some genetic alterations[76, 77]. Similar to that observed in inflammatory diseases, maintenance of the unique characteristics of each CAF subpopulation may be driven by epigenetic imprinting of tumor fibroblasts. Indeed, CAFs display distinct DNA methylation patterns, which maintain their pathological properties[78, 79]. Therefore, targeting CAFs by using epigenetic modifiers may be a promising tool to alter the CAF phenotype from immunosuppressive to immunostimulatory.

### Epigenetic regulation of cancer cells

Aberrant epigenetic changes contribute to the pathogenesis of various diseases, including cancer[80]. Aberrant DNA methylation has been shown to be involved in the initiation and progression of cancer. In particular, there is a global loss of DNA methylation in cancer cells along with simultaneous focal hypermethylation, particularly at promoter CpG islands[81–83]. Hypermethylation of CpG islands in tumor suppressor genes can lead to their silencing and ultimately contribute to cancer development. However, herein, we focused our discussion on epigenetic alterations that allow cancer cells to evade immune surveillance and rejection. The ability to evade destruction by the immune system is a cancer hallmark[84, 85]. Both innate and adaptive immune cells can engage in direct killing of cancer cells. Epigenetic mechanisms are critical for many processes in the so-called cancer–immunity cycle[86, 87]. Here, we discuss the impact of cancer cell-intrinsic epigenetic modifications. Immune cell-mediated killing requires cancer cells to express certain molecular features, including death receptors, stress-induced ligands, MHC-I molecules, intact antigen presentation machinery, and tumor-associated antigens. Immune cell-mediated cancer cell killing involves antigen-dependent and antigen-independent processes. The former are carried out primarily by CD8^+^ and CD4^+^ T cells, whereas the latter are executed primarily by NK cells[32, 88]. For T cells to effectively kill a cancer cell, the latter must process and present tumor-associated immunogenic peptides on its surface via MHC-I molecules. To do this, cancer cells must possess fully active antigen processing and presentation machinery (AgPPM). NK cell-mediated cancer cell killing is activated when a cancer cell is MHC-I negative and requires the cancer cell to express death receptors, such as the Fas ligand receptor and TNF receptor, and various stress-induced ligands on its surface[89]. Epigenetic processes play an important role in the ability of cancer cells to evade immune recognition and killing. For instance, DNA methylation and certain histone modifications contribute to silencing of various genes involved in antigen processing and presentation, as well as tumor-associated antigen genes, MHC class I and II genes and costimulatory molecule genes[90, 91]. In this regard, treatment of cancer cells with epigenetic therapies such as HDACis or HAT activators leads to upregulation of NK cell ligands [92] and AgPPM components, including tumor-associated antigens, cancer testis antigens, immunoproteasome subunits, peptide transporters, NLRC5, and MHC class I and II molecules[90]. For instance, colorectal cancer cells deficient in DNMTs show decreased methylation and increased expression of MHC class I genes and NK cell ligands [93]. Moreover, we found that two distinct DNA-damaging drugs, the platinoid oxaliplatin and the topoisomerase inhibitor mitoxantrone, strongly upregulate the MHC-I AgPPM in a manner dependent on the activation of NF-κB and p300/CBP through an incompletely understood mechanism dependent on the release of mitochondrial DNA. Ablation or inhibition of NF-κB and p300 prevented chemotherapy-induced MHC-I AgPPM upregulation, abrogating rejection of low MHC-I-expressing tumors by reinvigorating CD8^+^ CTLs. Both loss- and gain-of-function mutations in the human *EP300* locus were described to enhance tumor progression, suggesting context-dependent tumor-suppressive or oncogenic functions [94, 95]. *EP300* downregulation in human cancer was associated with reduced MHC-I AgPPM expression and changes in neoantigen amounts and presentation [90]. Collectively, these studies confirm that epigenetic processes play pivotal roles in the immune evasion capabilities of cancer cells.

Other epigenetic modifications resulting in DNA hypermethylation or hypomethylation in cancer cells, which are associated with overexpression of DNMTs and EZH2, particularly in melanoma patients, correlate with PD-L1 expression and T-cell function and control the response to immunotherapy [96]. EZH2 also regulates immunogenicity and antigen presentation[97].

## Concluding remarks and relevance to cancer prevention and treatment

**Fig. 1:**
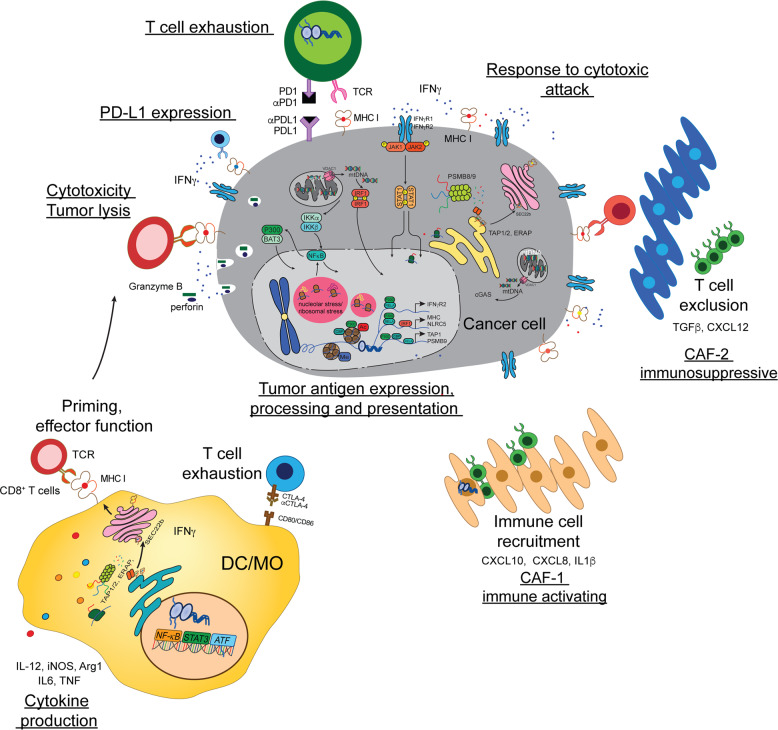
Major epigenetic regulation in antitumor immunity. Histone posttranslational modifications and DNA methylation play crucial roles in adaptive immune responses, including dendritic cell development, antitumor cytokine silencing or expression, and T-cell priming and activation. However, such modifications also control the exhausted phenotype in tumor-infiltrating CD8^+^ T cells. In cancer cells, histone and DNA modifications affect tumor antigen production, antigen processing and presentation machinery components and PD-L1 induction. Chromatin remodeling also regulates the response to cytotoxic attack in cancer cells. Epigenetic modifying agents (EMAs) can enhance multiple aspects of the antitumor immune response

Epigenetic silencing of immune-related genes is an important feature of the cancer genome that enables immune evasion during early tumor growth, metastatic dissemination, and acquisition of resistance to immunotherapies. This phenomenon impacts antigen processing and antigen presentation by malignant cells, while its reversal potentiates immunosurveillance and immune rejection, which would be useful in both cancer prevention and cancer treatment. Further modulation of the tumor microenvironment by altered expression of immunosuppressive cytokines impairs antigen-presenting cells and cytotoxic T-cell function. The potential reversal of immunosuppression by epigenetic modulation is a promising and versatile therapeutic approach to reinstate endogenous immune recognition and tumor lysis. Recent efforts have been made to determine whether epigenetic therapies can be used to enhance the efficacy of cancer immunotherapies. Drugs such as oxaliplatin and mitoxantrone or HDACis and HAT activators might be able to potentiate the response to immunotherapy. Epigenetic modifying compounds (EMCs) and chemotherapeutics can boost tumor antigen expression and endogenous antigen processing, increase the presentation of surface antigens by MHC molecules, and boost T-cell priming through increased expression of costimulatory molecules. EMCs may also enhance both cellular and cytokine-mediated effector T-cell functions and tumor lysis. EMCs can alter checkpoint inhibition targeting the PD-1/PD-L1 and CTLA-4/CD28 axes, resulting in more efficient effector T-cell function and subsequent tumor rejection. Table 1 provides a summary of ongoing clinical trials that combine histone modifiers and ICT [98, 99]. Most of these trials are still recruiting or not yet completed; thus, it is too early to conclude which combination will be the most effective in increasing antigen presentation and T-cell activation and supporting a strong antitumor immune response. Furthermore, changes in food composition, caloric restriction, and usage of particular metabolites that regulate histone modification [18–20] provide promising strategies for the treatment and prevention of cancer and improvement of immunotherapy. However, deeper mechanistic studies are needed to fully understand this complex interplay (Fig. 1).

**Table 1:** Clinical trials using histone modification in combination with immunotherapy, particularly immune checkpoint inhibitor therapy (ICIT)

Patient sample size: <5050–100101–200>200

*AML* acute myeloid lymphoma, *MDS* myelodysplastic syndrome, *NSCLC* non-small cell lung cancer, *CMML* chronic myelomonocytic leukemia, *CRC* colorectal cancer, *i* inhibitor, *DNMTs* DNA methyltransferases, *HDACs* histone deacetylases, *HMTs* histone methyltransferases, *HDM* histone demethylase, *BRD (BET)* bromodomain and extraterminal motif, *Pembrolizumab* anti-PD-1 monoclonal antibody, *durvalumab* anti-PD-L1 monoclonal antibody, *avelumab* anti-PD-L1 monoclonal antibody, *nivolumab* anti-PD-1 monoclonal antibody, *atezolizumab* anti-PD-1 monoclonal antibody, *OR* overall response, *PFS* progression-free survival, *PR* partial response, *SD* stable disease

## References

[1] Netea MG, Joosten LAB, Latz E, Mills KHG, Natoli G, Stunnenberg HG, et al. Trained immunity: a program of innate immune memory in health and disease. Science. 2016;352. 10.1126/science.aaf1098. 10.1126/science.aaf1098 PMC5087274 27102489

[2] Naik S, Larsen SB, Gomez NC, Alaverdyan K, Sendoel A, Yuan S (2017). Inflammatory memory sensitizes skin epithelial stem cells to tissue damage. Nature.

[3] Placek K, Schultze JL, Aschenbrenner AC (2019). Epigenetic reprogramming of immune cells in injury, repair, and resolution. J Clin Invest.

[4] Cosgrove MS, Boeke JD, Wolberger C (2004). Regulated nucleosome mobility and the histone code. Nat Struct Mol Biol.

[5] Akhtar A, Becker PB (2000). Activation of transcription through histone H4 acetylation by MOF, an acetyltransferase essential for dosage compensation in Drosophila. Mol Cell.

[6] Ren Q, Gorovsky MA (2001). Histone H2A.Z acetylation modulates an essential charge patch. Mol Cell.

[7] Tie F, Banerjee R, Stratton CA, Prasad-Sinha J, Stepanik V, Zlobin A (2009). CBP-mediated acetylation of histone H3 lysine 27 antagonizes drosophila polycomb silencing. Development.

[8] Yang X-J, Seto E (2007). HATs and HDACs: from structure, function and regulation to novel strategies for therapy and prevention. Oncogene.

[9] Cao R, Wang L, Wang H, Xia L, Erdjument-Bromage H, Tempst P (2002). Role of histone H3 lysine 27 methylation in Polycomb-group silencing. Science.

[10] Tessarz P, Kouzarides T (2014). Histone core modifications regulating nucleosome structure and dynamics. Nat Rev Mol Cell Biol.

[11] Kiefer JC (2007). Epigenetics in development. Developmental Dyn.

[12] Abu-Remaileh M, Bender S, Raddatz G, Ansari I, Cohen D, Gutekunst J (2015). Chronic inflammation induces a novel epigenetic program that is conserved in intestinal adenomas and in colorectal cancer. Cancer Res.

[13] Maekita T, Nakazawa K, Mihara M, Nakajima T, Yanaoka K, Iguchi M (2006). High levels of aberrant DNA methylation in Helicobacter pylori-infected gastric mucosae and its possible association with gastric cancer risk. Clin Cancer Res.

[14] Chiba T, Marusawa H, Ushijima T (2012). Inflammation-associated cancer development in digestive organs: mechanisms and roles for genetic and epigenetic modulation. Gastroenterology.

[15] Vanden Berghe W, Ndlovu’Matladi N, Hoya-Arias R, Dijsselbloem N, Gerlo S, Haegeman G (2006). Keeping up NF-κB appearances: epigenetic control of immunity or inflammation-triggered epigenetics. Biochemical Pharmacol.

[16] Zhang R, Kang KA, Kim KC, Na S-Y, Chang WY, Kim GY (2013). Oxidative stress causes epigenetic alteration of CDX1 expression in colorectal cancer cells. Gene.

[17] Maiuri AR, O’Hagan HM (2016). Interplay between inflammation and epigenetic changes in cancer. Prog Mol Biol Transl Sci.

[18] Molina-Serrano D, Kyriakou D, Kirmizis A (2019). Histone modifications as an intersection between diet and longevity. Front Genet.

[19] Delage B, Dashwood RH (2008). Dietary manipulation of histone structure and function. Annu Rev Nutr.

[20] Ferrere G, Alou MT, Liu P, Goubet A-G, Fidelle M, Kepp O, et al. Ketogenic diet and ketone bodies enhance the anticancer effects of PD-1 blockade. JCI Insight. 2021;6. 10.1172/jci.insight.145207. 10.1172/jci.insight.145207 PMC7934884 33320838

[21] Kominsky DJ, Campbell EL, Colgan SP (2010). Metabolic shifts in immunity and inflammation. J Immunol.

[22] Ng S-F, Lin RCY, Laybutt DR, Barres R, Owens JA, Morris MJ (2010). Chronic high-fat diet in fathers programs β-cell dysfunction in female rat offspring. Nature.

[23] Hermsdorff HH, Mansego ML, Campión J, Milagro FI, Zulet MA, Martínez JA (2013). TNF-alpha promoter methylation in peripheral white blood cells: relationship with circulating TNFα, truncal fat and n-6 PUFA intake in young women. Cytokine.

[24] Simar D, Versteyhe S, Donkin I, Liu J, Hesson L, Nylander V (2014). DNA methylation is altered in B and NK lymphocytes in obese and type 2 diabetic human. Metabolism.

[25] Demaria M, Giorgi C, Lebiedzinska M, Esposito G, D’Angeli L, Bartoli A (2010). A STAT3-mediated metabolic switch is involved in tumour transformation and STAT3 addiction. Aging (Albany NY).

[26] Johnson C, Warmoes MO, Shen X, Locasale JW (2015). Epigenetics and cancer metabolism. Cancer Lett.

[27] Bian Y, Li W, Kremer DM, Sajjakulnukit P, Li S, Crespo J (2020). Cancer SLC43A2 alters T cell methionine metabolism and histone methylation. Nature.

[28] Rasmussen KD, Helin K (2016). Role of TET enzymes in DNA methylation, development, and cancer. Genes Dev.

[29] Xu W, Yang H, Liu Y, Yang Y, Wang P, Kim S-H (2011). Oncometabolite 2-hydroxyglutarate is a competitive inhibitor of α-ketoglutarate-dependent dioxygenases. Cancer Cell.

[30] Peng M, Yin N, Chhangawala S, Xu K, Leslie CS, Li MO (2016). Aerobic glycolysis promotes T helper 1 cell differentiation through an epigenetic mechanism. Science.

[31] Zhang Q, Cao X (2019). Epigenetic regulation of the innate immune response to infection. Nat Rev Immunol.

[32] Maio M, Covre A, Fratta E, Di Giacomo AM, Taverna P, Natali PG (2015). Molecular pathways: at the crossroads of cancer epigenetics and immunotherapy. Clin Cancer Res.

[33] Gajewski TF, Schreiber H, Fu Y-X (2013). Innate and adaptive immune cells in the tumor microenvironment. Nat Immunol.

[34] Woo S-R, Corrales L, Gajewski TF (2015). Innate immune recognition of cancer. Annu Rev Immunol.

[35] Shalapour S, Karin M (2015). Immunity, inflammation, and cancer: an eternal fight between good and evil. J Clin Invest.

[36] Shalapour S, Karin M (2019). Pas de Deux: control of anti-tumor immunity by cancer-associated inflammation. Immunity.

[37] Coussens LM, Werb Z (2002). Inflammation and cancer. Nature.

[38] Ivashkiv LB (2013). Epigenetic regulation of macrophage polarization and function. Trends Immunol.

[39] Däbritz J, Menheniott TR (2014). Linking immunity, epigenetics, and cancer in inflammatory bowel disease. Inflamm Bowel Dis.

[40] Yang X, Wang X, Liu D, Yu L, Xue B, Shi H (2014). Epigenetic regulation of macrophage polarization by DNA methyltransferase 3b. Mol Endocrinol.

[41] Hardbower DM, Asim M, Luis PB, Singh K, Barry DP, Yang C (2017). Ornithine decarboxylase regulates M1 macrophage activation and mucosal inflammation via histone modifications. Proc Natl Acad Sci USA.

[42] Shen Y, Kapfhamer D, Minnella AM, Kim J-E, Won SJ, Chen Y (2017). Bioenergetic state regulates innate inflammatory responses through the transcriptional co-repressor CtBP. Nat Commun.

[43] Yeung F, Hoberg JE, Ramsey CS, Keller MD, Jones DR, Frye RA (2004). Modulation of NF-kappaB-dependent transcription and cell survival by the SIRT1 deacetylase. EMBO J.

[44] Misawa T, Takahama M, Kozaki T, Lee H, Zou J, Saitoh T (2013). Microtubule-driven spatial arrangement of mitochondria promotes activation of the NLRP3 inflammasome. Nat Immunol.

[45] Wilson CB, Rowell E, Sekimata M (2009). Epigenetic control of T-helper-cell differentiation. Nat Rev Immunol.

[46] Schoenborn JR, Dorschner MO, Sekimata M, Santer DM, Shnyreva M, Fitzpatrick DR (2007). Comprehensive epigenetic profiling identifies multiple distal regulatory elements directing transcription of the gene encoding interferon-gamma. Nat Immunol.

[47] Dispirito JR, Shen H (2010). Histone acetylation at the single-cell level: a marker of memory CD8+ T cell differentiation and functionality. J Immunol.

[48] Ballas ZK (1984). The use of 5-azacytidine to establish constitutive interleukin 2-producing clones of the EL4 thymoma. J Immunol.

[49] Young HA, Ghosh P, Ye J, Lederer J, Lichtman A, Gerard JR (1994). Differentiation of the T helper phenotypes by analysis of the methylation state of the IFN-gamma gene. J Immunol.

[50] Chen G-Y, Osada H, Santamaria-Babi LF, Kannagi R (2006). Interaction of GATA-3/T-bet transcription factors regulates expression of sialyl Lewis X homing receptors on Th1/Th2 lymphocytes. Proc Natl Acad Sci USA.

[51] Ivanov II, Zhou L, Littman DR (2007). Transcriptional regulation of Th17 cell differentiation. Semin Immunol.

[52] Waibel M, Christiansen AJ, Hibbs ML, Shortt J, Jones SA, Simpson I (2015). Manipulation of B-cell responses with histone deacetylase inhibitors. Nat Commun.

[53] Raghuraman S, Donkin I, Versteyhe S, Barrès R, Simar D (2016). The emerging role of epigenetics in inflammation and immunometabolism. Trends Endocrinol Metab.

[54] Guo M, Price MJ, Patterson DG, Barwick BG, Haines RR, Kania AK (2018). EZH2 represses the B cell transcriptional program and regulates antibody-secreting cell metabolism and antibody production. J Immunol.

[55] Béguelin W, Popovic R, Teater M, Jiang Y, Bunting KL, Rosen M (2013). EZH2 is required for germinal center formation and somatic EZH2 mutations promote lymphoid transformation. Cancer Cell.

[56] Stairiker CJ, Thomas GD, Salek-Ardakani S. EZH2 as a regulator of CD8+ T cell fate and function. Front Immunol. 2020;11. 10.3389/fimmu.2020.593203. 10.3389/fimmu.2020.593203 PMC7574680 33117406

[57] Goswami S, Apostolou I, Zhang J, Skepner J, Anandhan S, Zhang X (2018). Modulation of EZH2 expression in T cells improves efficacy of anti–CTLA-4 therapy. J Clin Invest.

[58] Karantanos T, Christofides A, Bardhan K, Li L, Boussiotis VA. Regulation of T cell differentiation and function by EZH2. Front Immunol. 2016;7. 10.3389/fimmu.2016.00172. 10.3389/fimmu.2016.00172 PMC4853381 27199994

[59] Philip M, Fairchild L, Sun L, Horste EL, Camara S, Shakiba M (2017). Chromatin states define tumour-specific T cell dysfunction and reprogramming. Nature.

[60] Mariathasan S, Turley SJ, Nickles D, Castiglioni A, Yuen K, Wang Y (2018). TGFβ attenuates tumour response to PD-L1 blockade by contributing to exclusion of T cells. Nature.

[61] Davidson S, Coles M, Thomas T, Kollias G, Ludewig B, Turley S, et al. Fibroblasts as immune regulators in infection, inflammation and cancer. Nat Rev Immunol. 2021;1–14. 10.1038/s41577-021-00540-z. 10.1038/s41577-021-00540-z 33911232

[62] Kalluri R (2016). The biology and function of fibroblasts in cancer. Nat Rev Cancer.

[63] Chen Y, Kim J, Yang S, Wang H, Wu C-J, Sugimoto H (2021). Type I collagen deletion in αSMA+ myofibroblasts augments immune suppression and accelerates progression of pancreatic cancer. Cancer Cell.

[64] Costa A, Kieffer Y, Scholer-Dahirel A, Pelon F, Bourachot B, Cardon M (2018). Fibroblast heterogeneity and immunosuppressive environment in human breast cancer. Cancer Cell.

[65] Erez N, Truitt M, Olson P, Arron ST, Hanahan D (2010). Cancer-associated fibroblasts are activated in incipient neoplasia to orchestrate tumor-promoting inflammation in an NF-kappaB-dependent manner. Cancer Cell.

[66] Kieffer Y, Hocine HR, Gentric G, Pelon F, Bernard C, Bourachot B (2020). Single-cell analysis reveals fibroblast clusters linked to immunotherapy resistance in. Cancer Cancer Disco.

[67] Ammirante M, Shalapour S, Kang Y, Jamieson CAM, Karin M (2014). Tissue injury and hypoxia promote malignant progression of prostate cancer by inducing CXCL13 expression in tumor myofibroblasts. Proc Natl Acad Sci USA.

[68] Shalapour S, Lin X-J, Bastian IN, Brain J, Burt AD, Aksenov AA (2017). Inflammation-induced IgA+ cells dismantle anti-liver cancer immunity. Nature.

[69] Shalapour S, Font-Burgada J, Di Caro G, Zhong Z, Sanchez-Lopez E, Dhar D (2015). Immunosuppressive plasma cells impede T-cell-dependent immunogenic chemotherapy. Nature.

[70] Biffi G, Oni TE, Spielman B, Hao Y, Elyada E, Park Y (2019). IL1-Induced JAK/STAT signaling is antagonized by TGFβ to shape CAF heterogeneity in pancreatic ductal adenocarcinoma. Cancer Disco.

[71] Becker LM, O’Connell JT, Vo AP, Cain MP, Tampe D, Bizarro L (2020). Epigenetic reprogramming of cancer-associated fibroblasts deregulates glucose metabolism and facilitates progression of breast cancer. Cell Rep.

[72] Bhagat TD, Von Ahrens D, Dawlaty M, Zou Y, Baddour J, Achreja A (2019). Lactate-mediated epigenetic reprogramming regulates formation of human pancreatic cancer-associated fibroblasts. eLife.

[73] Lee YT, Tan YJ, Falasca M, Oon CE. Cancer-associated fibroblasts: epigenetic regulation and therapeutic intervention in breast cancer. Cancers (Basel). 2020;12. 10.3390/cancers12102949. 10.3390/cancers12102949 PMC7600259 33066013

[74] Mishra R, Haldar S, Suchanti S, Bhowmick NA (2019). Epigenetic changes in fibroblasts drive cancer metabolism and differentiation. Endocr Relat Cancer.

[75] Zhou Y, Bian S, Zhou X, Cui Y, Wang W, Wen L (2020). Single-cell multiomics sequencing reveals prevalent genomic alterations in tumor stromal cells of human colorectal cancer. Cancer Cell.

[76] Shalapour S, Eckert C, Seeger K, Pfau M, Prada J, Henze G (2010). Leukemia-associated genetic aberrations in mesenchymal stem cells of children with acute lymphoblastic leukemia. J Mol Med (Berl).

[77] Menendez P, Catalina P, Rodríguez R, Melen GJ, Bueno C, Arriero M (2009). Bone marrow mesenchymal stem cells from infants with MLL-AF4+ acute leukemia harbor and express the MLL-AF4 fusion gene. J Exp Med.

[78] Xiao Q, Zhou D, Rucki AA, Williams J, Zhou J, Mo G (2016). Cancer-associated fibroblasts in pancreatic cancer are reprogrammed by tumor-induced alterations in genomic DNA methylation. Cancer Res.

[79] Albrengues J, Bertero T, Grasset E, Bonan S, Maiel M, Bourget I (2015). Epigenetic switch drives the conversion of fibroblasts into proinvasive cancer-associated fibroblasts. Nat Commun.

[80] Baylin SB, Jones PA. Epigenetic determinants of cancer. Cold Spring Harb Perspect Biol. 2016;8. 10.1101/cshperspect.a019505. 10.1101/cshperspect.a019505 PMC5008069 27194046

[81] Berman BP, Weisenberger DJ, Aman JF, Hinoue T, Ramjan Z, Liu Y (2011). Regions of focal DNA hypermethylation and long-range hypomethylation in colorectal cancer coincide with nuclear lamina-associated domains. Nat Genet.

[82] Hansen KD, Timp W, Bravo HC, Sabunciyan S, Langmead B, McDonald OG (2011). Increased methylation variation in epigenetic domains across cancer types. Nat Genet.

[83] Zelic R, Fiano V, Zugna D, Grasso C, Delsedime L, Daniele L (2016). Global hypomethylation (LINE-1) and gene-specific hypermethylation (GSTP1) on initial negative prostate biopsy as markers of prostate cancer on a rebiopsy. Clin Cancer Res.

[84] Hanahan D, Weinberg RA (2000). The hallmarks of cancer. Cell.

[85] Hanahan D, Weinberg RA (2011). Hallmarks of cancer: the next generation. Cell.

[86] Chen DS, Mellman I (2013). Oncology meets immunology: the cancer-immunity cycle. Immunity.

[87] Chen DS, Mellman I (2017). Elements of cancer immunity and the cancer-immune set point. Nature.

[88] Jhunjhunwala S, Hammer C, Delamarre L (2021). Antigen presentation in cancer: insights into tumour immunogenicity and immune evasion. Nat Rev Cancer.

[89] Waldhauer I, Steinle A (2008). NK cells and cancer immunosurveillance. Oncogene.

[90] Zhou Y, Bastian IN, Long MD, Dow M, Li W, Liu T, et al. Activation of NF-κB and p300/CBP potentiates cancer chemoimmunotherapy through induction of MHC-I antigen presentation. PNAS. 2021;118. 10.1073/pnas.2025840118. 10.1073/pnas.2025840118 PMC7923353 33602823

[91] Sigalotti L, Fratta E, Coral S, Maio M (2014). Epigenetic drugs as immunomodulators for combination therapies in solid tumors. Pharm Ther.

[92] Zhang C, Wang Y, Zhou Z, Zhang J, Tian Z (2009). Sodium butyrate upregulates expression of NKG2D ligand MICA/B in HeLa and HepG2 cell lines and increases their susceptibility to NK lysis. Cancer Immunol Immunother.

[93] Sers C, Kuner R, Falk CS, Lund P, Sueltmann H, Braun M (2009). Down-regulation of HLA Class I and NKG2D ligands through a concerted action of MAPK and DNA methyltransferases in colorectal cancer cells. Int J Cancer.

[94] Attar N, Kurdistani SK. Exploitation of EP300 and CREBBP lysine acetyltransferases by cancer. Cold Spring Harb Perspect Med. 2017;7. 10.1101/cshperspect.a026534. 10.1101/cshperspect.a026534 PMC5334244 27881443

[95] Gayther SA, Batley SJ, Linger L, Bannister A, Thorpe K, Chin S-F (2000). Mutations truncating the EP300 acetylase in human cancers. Nat Genet.

[96] Emran AA, Chatterjee A, Rodger EJ, Tiffen JC, Gallagher SJ, Eccles MR (2019). Targeting DNA methylation and EZH2 activity to overcome melanoma resistance to immunotherapy. Trends Immunol.

[97] Zingg D, Arenas-Ramirez N, Sahin D, Rosalia RA, Antunes AT, Haeusel J (2017). The histone methyltransferase Ezh2 controls mechanisms of adaptive resistance to tumor immunotherapy. Cell Rep.

[98] Cheng Y, He C, Wang M, Ma X, Mo F, Yang S (2019). Targeting epigenetic regulators for cancer therapy: mechanisms and advances in clinical trials. Sig Transduct Target Ther.

[99] Villanueva L, Álvarez-Errico D, Esteller M (2020). The contribution of epigenetics to cancer immunotherapy. Trends Immunol.

